# Real-time detection of plant leaf diseases based on improved YOLOv13-LM in complex field environments

**DOI:** 10.3389/fpls.2026.1819646

**Published:** 2026-06-15

**Authors:** Tong Li, Jiangtao Su, Shuang Li

**Affiliations:** 1 College of Agriculture and Forestry Economics and Management, Lanzhou University of Finance and Economics, Lanzhou, China; 2 Institute of Semiconductors, Chinese Academy of Sciences, Beijing, China

**Keywords:** crop health monitoring, deep learning, object recognition, plant leaf disease detection, sustainable pest management, YOLOv13-LM

## Abstract

Plant leaf diseases are a major factor leading to crop yield reduction, seriously threatening food security and sustainable agricultural development. Timely and accurate detection is crucial for scientific prevention and control. Traditional manual detection is time-consuming, labor-intensive, and highly subjective. Existing deep learning detection models still have significant shortcomings in complex field scenarios, struggling to balance detection accuracy, real-time performance, and stability. They are easily affected by background interference, differences in lesion scale, and sample imbalance, and some models are not lightweight enough to meet the needs of real-time field detection. Therefore, this paper uses YOLOv13 as the baseline model and constructs a YOLOv13-LM model through multi-module collaborative optimization. The optimization directions cover the backbone, neck, detection head, and loss function, strengthening lesion feature extraction and multi-scale fusion, reducing task interference, and improving localization accuracy. Model validation was completed in a complex farmland environment. The results show that the model’s mAP@0.5 is improved by 5.4 percentage points to 87.9% compared to the original YOLOv13, the FPS is improved by 21.1% to 46 frames/second, and the number of parameters and computational cost are reduced by 27.3% and 25.7% respectively. The overall performance is better than the mainstream YOLO models of the same scale. However, the lightweight nature of the model is still not as good as that of the ultra-lightweight model, and its generalization and interpretability need to be improved. In the future, we will focus on ultra-lightweight design, expanding the generalization ability of multiple crops and diseases, and studying the interpretability of the model to further adapt to the actual needs of field applications.

## 1 Introduction

Plant leaf diseases are a significant factor causing global crop yield reductions, directly threatening food security and sustainable agricultural development (Harakannanavar et al., 2022). Timely and accurate disease detection is crucial for scientific prevention and control, reducing yield losses, and minimizing pesticide use (Abd Algani et al., 2023). Traditional leaf disease detection relies mainly on manual visual recognition, which is inefficient, highly subjective, and dependent on professional experience, making it difficult to meet the needs of large-scale field inspections and the rapid identification of small lesions in the early stages (Khan et al., 2023; Du et al., 2025; Zhou et al., 2025). With the development of artificial intelligence and computer vision technologies, deep learning-based object detection methods provide a feasible path for automatic recognition of plant leaf diseases (Dahiya et al., 2022; Zhang et al., 2025). Among them, YOLO series models have been widely applied in disease detection due to their balanced performance in detection speed and accuracy.

Currently, significant progress has been made in optimizing leaf disease detection based on the YOLO series, but there remains a core issue of balancing detection accuracy and inference speed in complex field environments (Ponnusamy et al., 2025; Tao et al., 2025). To improve accuracy, many improvement methods deepen the network or introduce complex attention mechanisms to enhance feature extraction, leading to a dramatic increase in parameters and computational cost, thereby slowing down inference speed (Soeb et al., 2023; Kaur et al., 2025; Long et al., 2026). These methods are unsuitable for scenarios such as drone inspections and real-time detection on mobile devices. Lightweight models, while ensuring speed, often sacrifice feature extraction accuracy (Mathew and Mahesh, 2022; Lu et al., 2025). In conditions with lighting variations, leaf occlusion, complex backgrounds like weeds and soil, and significant differences in lesion scales, these models are prone to false negatives and false positives.

**Figure 1:**
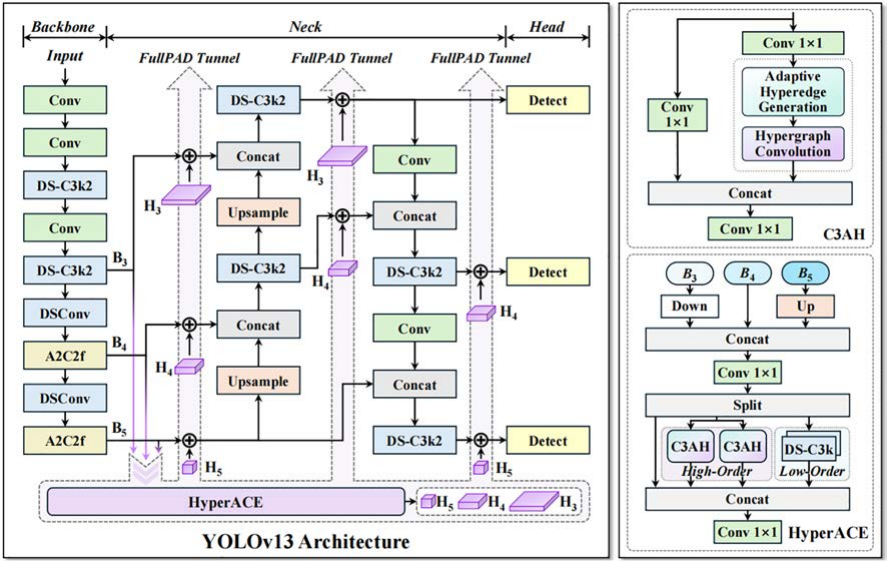
YOLOv13 model architecture diagram (including Backbone feature extraction module, HyperACE correlation enhancement module, FullPAD feature distribution paradigm, Neck multi-scale fusion module, and Head detection head) (Lei et al., 2025).

YOLOv13(**Figure 1**), the latest model in the YOLO series, introduces the Hypergraph Adaptive Correlation Enhancement (HyperACE) mechanism and the Full Pipeline Aggregation and Distribution (FullPAD) paradigm, effectively enhancing high-order feature representation capability (Ramos and Sappa, 2025). Combined with the DS-C3k2 lightweight module to reduce computational overhead, it performs well in general object detection tasks (Wang et al., 2025). However, when directly applied to complex field leaf disease detection, several issues remain: First, the HyperACE and FullPAD structures introduce additional computational overhead, making real-time performance insufficient for field deployment requirements (Begum et al., 2025; Lei et al., 2025). Second, the DS-C3k2 module in the Backbone is not specifically optimized for separating lesions from the background, making the features vulnerable to interference (Zhang et al., 2025; Tang et al., 2026; Wang et al., 2026). Third, the SPPF module in the Neck has redundant pooling branches, and the multi-scale lesion feature fusion efficiency is insufficient (Ning et al., 2026). Fourth, the coupled detection head causes interference between classification and regression tasks, and the original loss function has limited optimization accuracy for locating small lesions, limiting the model’s performance in practical field scenarios (Ramos and Sappa, 2025).

Considering the baseline model selection principles comprehensively, this paper does not directly use original lightweight detection models such as YOLOv10-S and YOLOv12-S as the research basis. Limited by the extremely minimalist network architecture, such lightweight models have inherent bottlenecks in high-level feature modeling and fine-grained mining of tiny and blurred disease spots in complex farmland environments, with limited room for optimization and improvement. In contrast, YOLOv13 relies on the special structures of HyperACE and FullPAD, and possesses significantly better feature representation capability than lightweight models of the same level, which can fully capture the deep semantic features of field diseases and multi-scale disease spot information, better meeting the high-precision detection requirements under complex field backgrounds. Although the original YOLOv13 has defects of computational redundancy and insufficient real-time performance, its excellent feature learning potential has extremely high renovation value. Therefore, this paper retains the backbone feature extraction architecture of YOLOv13, and carries out lightweight reconstruction and structural optimization targeting its computational redundancy and scene adaptation defects. While retaining the advantage of high-precision feature extraction, it makes up for the shortcoming of real-time performance. Combined with the scene characteristics of field leaf disease detection, the YOLOv13-LM model is proposed through multi-module collaborative optimization design, aiming to achieve an accurate balance between detection accuracy and inference speed and meet the actual field detection requirements.

1. Lightweight enhancement improvement is carried out on the Backbone module. On the basis of retaining the core structure of the DS-C3k2 module, the convolution therein is replaced with the optimized Depthwise Separable Convolution (DWConv) to further reduce computational redundancy. Meanwhile, the ECA lightweight attention module is embedded to strengthen lesion feature responses and suppress background interference without significantly increasing the computation amount, achieving dual improvements in lightweight design and feature extraction accuracy;
2. The structure of the Neck module is optimized: redundant pooling branches of the SPPF module are simplified to reduce invalid computations, and a Multi-Scale Convolution (MSC) module is introduced to enhance the fusion capability of feature information of lesions at different scales, adapting to the scenario characteristic of coexisting tiny and large-area lesions in the field;
3. The detection head and loss function are improved: a lightweight decoupled detection head is designed to separate classification and regression branches, reducing task interference. Additionally,

a combination of EIoU loss and lightweight Focal Loss is adopted, which not only improves the localization accuracy of tiny lesions but also effectively solves the problem of sample category imbalance in leaf diseases, balancing detection performance and computational efficiency.

The subsequent sections of this paper will first introduce the dataset used in the experiments and its preprocessing methods, elaborate on the original architecture of YOLOv13, the improvement details of the proposed YOLOv13-LM model, and the training strategy. Then, the advantages of the model in detection accuracy and real-time performance are systematically verified through ablation experiments, comparative experiments, and visualization results. Finally, an in-depth discussion of the experimental results is presented, and the research conclusions of the full paper are summarized.

## 2 Materials and methods

### 2.1 Datasets and preprocessing

**Figure 2:**
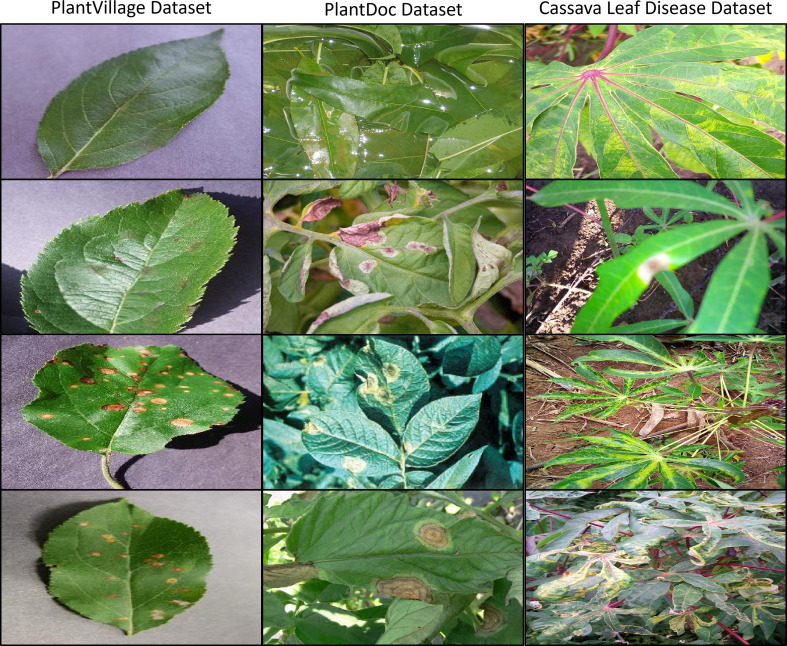
Typical samples from the PlantDoc, PlantVillage, and Cassava Leaf Disease datasets (including healthy leaves, multi-crop and multi-disease types, varying lesion scales, and complex detection scenes with field weeds/soil background, leaf occlusion/curling, and uneven lighting).

To validate the performance of YOLOv13-LM in complex field environments for leaf disease detection, this study conducts experiments using three internationally recognized benchmark datasets: PlantDoc, PlantVillage, and Cassava Leaf Disease Dataset. All datasets are publicly available and can be directly downloaded from the official links, ensuring the reproducibility and transparency of the experiments. The selected datasets cover a variety of crop types, different disease severities, and complex real-world field scenarios, effectively simulating issues such as lighting variations, leaf occlusion, soil and weed interference, and large lesion scale differences in real-world detection. This ensures that the experimental results are both reliable and generalizable, as shown in **Table 1** and **Figure 2**.

**Table 1:** Overview of the PlantDoc dataset, PlantVillage dataset, and cassava leaf disease dataset.

Dataset	Crop type	Disease categories	Total images	Annotated objects	Scene features
PlantDoc Dataset (Singh et al., 2020)	14 crops including apple, tomato and maize	27 classes	2598	6243 lesions	Real field scenes, leaf occlusion, soil/weeds interference, uneven illumination, large variation in lesion scale.
PlantVillage Dataset (Noyan, 2022)	38 crops including tomato, potato and grape	54 classes (including healthy)	54306	128542 lesions	Laboratory and natural field scenes, sufficient samples, clear features, minor noise.
Cassava Leaf Disease Dataset (Zhong et al., 2022)	Cassava	6 classes (5 diseases + 1 healthy)	21397	58761 lesions	Real African field scenes, drastic illumination changes, leaf curling, cluttered background.

The original resolution of all experimental images ranges from 300×300 to 4032×3024. During preprocessing, all images are uniformly scaled to the model input size of 640×640 pixels, and pixel normalization to the range [0, 1] is performed to speed up training convergence. To address issues such as sample distribution imbalance, strong scene interference, and inconsistent image sizes, a standardized preprocessing workflow is applied. First, blurry, damaged, mislabeled, and duplicate samples are manually removed to ensure the quality of the training data. Next, data augmentation strategies such as random cropping, brightness and contrast perturbations, horizontal flipping, and Gaussian noise addition are applied to expand the training set, alleviate class imbalance, and improve the model’s robustness in complex field environments. All datasets are strictly divided into training, validation, and test sets in an 8:1:1 ratio for model parameter updates, hyperparameter tuning, and final performance evaluation. The LabelImg annotation tool is used to annotate the bounding boxes, following the YOLO format specification and the “complete enclosure, precise fitting” annotation protocol to ensure consistent and accurate class and location annotations, providing reliable data support for model training and performance verification.

### 2.2 Proposed YOLOv13-LM model

**Figure 3:**
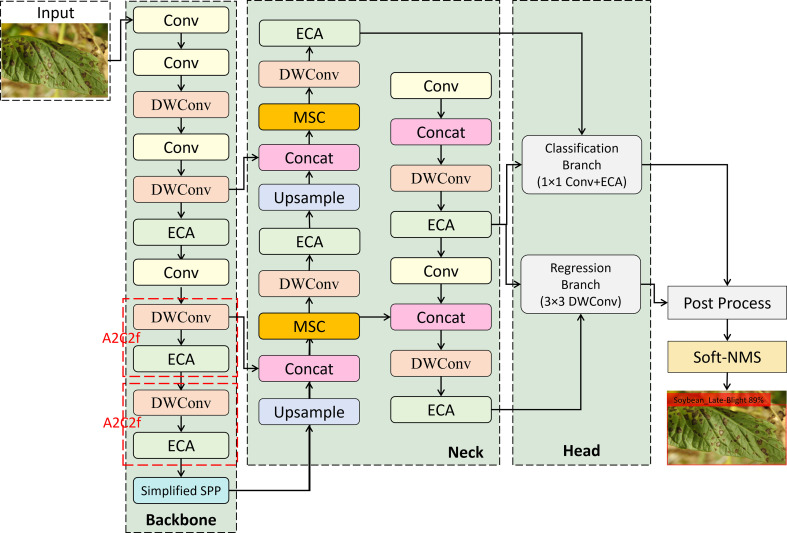
Overall architecture diagram of the YOLOv13-LM model (including DWConv+ECA lightweight feature extraction Backbone, simplified SPP+MSC multi-scale fusion Neck, lightweight decoupled detection Head, and EIoU+lightweight Focal Loss loss calculation module).

Although the original YOLOv13 model has strong high-order correlation modeling capabilities, it still struggles to balance accuracy and speed in complex field leaf disease detection. While HyperACE and FullPAD enhance feature representation, they also introduce additional computational overhead, and the original structure is not specifically optimized for separating lesion features from background interference in field scenarios. To address these issues, this paper proposes the YOLOv13-LM model, which collaborates in optimizing the Backbone, Neck, Head, and loss function across four dimensions: feature extraction efficiency, multi-scale fusion adaptability, task-specific detection, and loss calculation accuracy. This allows the model to better meet the real-time detection requirements in field scenarios. The overall architecture is shown in **Figure 3**, which clearly presents the sequential logic and data flow of the improved modules.

#### 2.2.1 DWConv+ECA module

**Figure 4:**
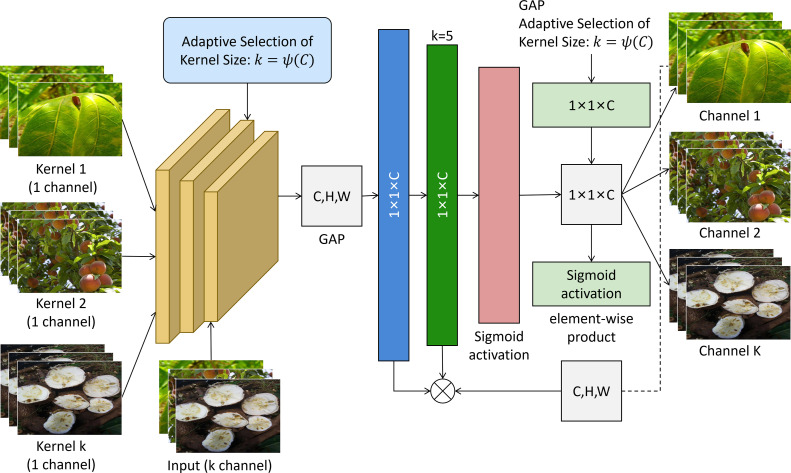
Diagram illustrating the improved Backbone architecture of YOLOv13-LM (depicting the sequential processing logic and feature flow of depthwise separable convolutions combined with ECA attention mechanisms).

The original YOLOv13 Backbone uses DS-C3k2 as the core module. While it achieves some degree of lightweight design through depthwise separable convolution, it has not been optimized for distinguishing lesions from the background in field scenarios. Additionally, the feature extraction process involves redundant channel-wise computations, making it difficult to meet the low-latency requirements for real-time detection (Lin et al., 2025; Zhanfang and Tuo, 2025). To reduce computational overhead and enhance lesion feature responses, this paper replaces the depthwise convolution in DS-C3k2 with an optimized depthwise separable convolution (DWConv), and embeds a lightweight ECA attention module after each DWConv layer, constructing a DWConv+ECA feature extraction unit, as shown in **Figure 4**.

Depthwise separable convolutions offer a key benefit by decomposing traditional standard convolution into two distinct sequential operations: depth convolution (also referred to as per-channel convolution) and point convolution (1×1 convolution). In depth convolution, each individual input channel is processed separately with dedicated convolution kernels, which only capture spatial feature information within that single channel. In contrast, point convolution leverages 1×1 convolution kernels to fuse feature information across different channels, thereby adjusting the channel dimensionality of the feature map. The computational complexity of DWConv is quantified by the Equation 1:

(1) $${\text{FLOPs}}_{\text{DWConv}}=H\times W\times C_{\text{in}}\times k^{2}+H\times W\times C_{\text{in}}\times C_{\text{out}}$$

In this formula, *H* and *W* denote the height and width of the input feature map, *C*_in_ stands for the count of input channels, *C*_out_ is the number of output channels, and *k* is the convolution kernel size (set to 3 in our experiments). In comparison to the convolution operations employed in the DS-C3k2 module of the original YOLOv13, DWConv breaks down inter-channel redundant connections more thoroughly, reducing the overall computational cost to $\frac{1}{C_{out}}+\frac{1}{k^{2}}$. For instance, when *C*_out_ is set to 64, the computational cost is reduced to roughly one-ninth of that of the original module, leading to a notable improvement in the model’s inference speed.

To offset the feature degradation induced by lightweight design—particularly in complex field environments where lesion features are prone to being obscured by background factors such as soil and weeds—the ECA lightweight attention module is integrated right after the DWConv layer. Unlike conventional attention mechanisms that rely on dimensionality reduction, the ECA module learns channel-wise weights adaptively via global average pooling (GAP) and 1D convolution, enabling targeted enhancement of lesion-related features. The process of computing these channel weights is outlined below: First, the feature map *X* (with dimensions *C*_in_ ×*H* ×*W*) generated by DWConv undergoes global average pooling, yielding the global feature for each channel as $\text{GAP}\left(X_{c}\right)=\frac{1}{H\times W}\sum_{i=1}^{H}\sum_{j=1}^{W}X_{c}\left(i,j\right)$ (where *X_c_*represents the feature map of the *c*-th channel). Next, 1D convolution is applied, with its kernel size adaptively calculated based on the number of input channels *C*_in_ using the formula $k=\log_{2}(C_{\text{in}})+1$ (an odd number is selected to ensure symmetry). This step captures local interdependencies across 164 different channels. Finally, the channel weight *ω_c_* is generated via the Sigmoid activation function, with 165 the calculation expressed as Equation 2:

(2) $$\omega_{c}=\sigma \left({\text{Conv}}_{1D}\left(\text{GAP}\left(X_{c}\right)\right)\right)$$

Here, *σ* denotes the Sigmoid activation function, which maps the weight values to the range of [0,1]. The enhanced feature map ${X^{\prime }}_{c}=\omega_{c}\cdot X_{c}$ is then obtained by performing element-wise multiplication between the learned channel weights and the original feature map. This approach effectively amplifies the feature response of lesion regions while suppressing irrelevant background interference, addressing the complex field background challenges that the original YOLOv13 failed to fully address—all without introducing a substantial increase in computational complexity.

#### 2.2.2 Simplified SPP+MSC module

**Figure 5:**
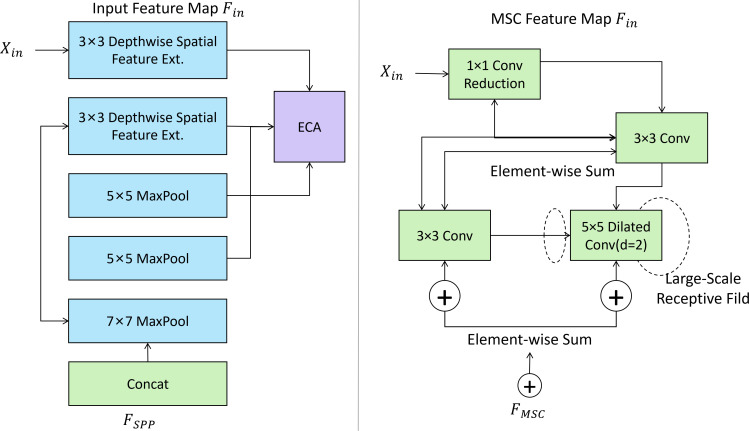
Schematic diagram of the YOLOv13-LM Neck improvement structure (showing the pooling branches of the simplified SPP and the multi-scale convolution fusion logic of MSC).

The original YOLOv13 Neck is based on the PAFPN structure and utilizes the FullPAD paradigm for enhanced feature distribution. However, its SPPF module employs a 4-branch pooling, where the 13×13 branch exceeds the main scale of field lesions, leading to redundant computations (Mao et al., 2025). Additionally, PAFPN’s cross-scale fusion relies solely on simple upsampling, downsampling, and concatenation, resulting in insufficient feature fusion capabilities for small, medium, and large lesions (Wang et al., 2022). To address these issues, this paper performs dual optimization on the Neck: simplifying the SPPF to a 3-branch pooling structure and adding a multi-scale convolution (MSC) module at the PAFPN fusion node, forming the simplified SPP+MSC multi-scale fusion architecture, as shown in **Figure 5**.

The simplified SPP module retains the core design of the original SPPF, but removes the redundant 13×13 pooling branch. It keeps the 3×3, 5×5, and 7×7 pooling branches, covering the main scale range of plant leaf diseases, from small lesions (2–10 pixels) to large-area lesions (50–200 pixels), while reducing unnecessary computations. The feature fusion process is as follows: the input feature map ***F***_in_ is subjected to 3 different scales of max pooling operations, and then concatenated with the original input feature map along the channel dimension to obtain the multi-scale fused feature map ***F***_SPP_. (Equation 3):

(3) $$F_{\text{SPP}}=\text{Concat}\left(F_{\text{in}},{\text{MaxPool}}_{33}\left(F_{\text{in}}\right),{\text{MaxPool}}_{55}\left(F_{\text{in}}\right),{\text{MaxPool}}_{77}\left(F_{\text{in}}\right)\right)$$

where Concat represents the concatenation operation along the channel dimension. By fusing features from different pooling scales, the model’s ability to express multi-scale lesions is enhanced.

To further improve the fusion efficiency of cross-scale features, the MSC module is added at the top-down and bottom-up fusion nodes of PAFPN. The module adopts a “1×1 convolution for dimensionality reduction + multi-scale convolution for parallel feature extraction” design: First, the input feature map ***F***_in_ is reduced to half of its original channel number using a 1×1 convolution to reduce subsequent computations. Then, 3×3 standard convolution (for capturing medium-scale lesion features) and 5×5 dilated convolution (dilation rate *d* = 2, enlarging receptive field to capture large-area lesion features) are used in parallel for feature extraction. Finally, the output features from both branches are element-wise added to obtain the multi-scale fused feature map ***F***_MSC_. (Equation 4):

(4) $$F_{\text{MSC}}={\text{Conv}}_{33}\left({\text{Conv}}_{11}\left(F_{\text{in}}\right)\right)+{\text{Conv}}_{55,d=2}\left({\text{Conv}}_{11}\left(F_{\text{in}}\right)\right)$$

This design eliminates the need for additional feature concatenation operations. Through the complementarity of the multi-scale convolution kernels, it effectively improves the fusion quality of features from different scales, especially for the case where small and large lesions coexist in field scenarios. This significantly reduces the miss detection rate and compensates for the original YOLOv13’s insufficient adaptability to multi-scale lesions.

#### 2.2.3 Loss function

The original YOLOv13 uses a coupled detection head where classification and regression tasks share feature branches, leading to issues such as task interference, excessively high convolution kernel dimensions, and increased inference latency (Kumar and Dhanalakshmi, 2024; Xu et al., 2024). Additionally, the original loss function is not optimized for the scale of field leaf lesions and sample distribution characteristics, making it sensitive to small lesion localization errors and computationally expensive (Sun et al., 2024; Li et al., 2025). To address these issues, this paper modifies the detection head to a lightweight decoupled structure and adopts a loss function that combines EIoU loss with lightweight Focal Loss, thereby improving both detection accuracy and speed.

The core design of the lightweight decoupled Head is to separate the classification and regression tasks into two independent branches, each focusing on extracting category and localization features, respectively. The classification branch uses a “1×1 convolution + ECA attention” lightweight structure. The 1×1 convolution reduces the input feature map’s channel number from 512 to 256, reducing computational load, while the ECA attention module enhances feature responses related to lesion categories. Finally, the Softmax activation function outputs the probability distribution for each class. The regression branch uses 3×3 depthwise convolutions to capture spatial localization information, outputting the bounding box offsets and confidence scores. By decoupling the tasks into separate branches, we effectively reduce interference between classification and localization features. Additionally, to adapt to the scale distribution of plant leaf diseases, the number of anchor boxes is re-clustered, reducing the original 9 anchors to 6 (to fit small, medium, and large lesions), which further reduces the computational cost of anchor box matching and adapts to the scale distribution of field leaf diseases.

For the loss function design, we first replace the regression loss in the original YOLOv13 loss function with EIoU loss. The EIoU loss, based on the CIoU overlap loss and center loss, adds width and height loss terms to more accurately optimize bounding box size matching, especially for fine-grained localization of small lesions. The formula is as Equation 5:

(5) $${\text{Loss}}_{\text{EIoU}}=1-\text{IoU}+\frac{\rho^{2}\left(b,b_{gt}\right)}{c^{2}}+\frac{\rho^{2}\left(w,w_{gt}\right)}{W^{2}}+\frac{\rho^{2}\left(h,h_{gt}\right)}{H^{2}}$$

where IoU is the intersection over union between the predicted and ground truth boxes, ***b*** and ***b***_gt_ are the center coordinates of the predicted and ground truth boxes, *ρ* is the Euclidean distance, *c* is the diagonal length of the smallest rectangle enclosing both the predicted and ground truth boxes, *w* and *h* are the width and height of the predicted box, and *w*_gt_ and *h*_gt_ are the width and height of the ground truth box. The introduction of the width and height loss terms enables EIoU loss to more directly optimize bounding box dimension errors, improving small lesion localization accuracy and compensating for the original model’s shortcomings in small object localization.

Aiming at the class imbalance problem in the leaf disease dataset (e.g., high proportion of common disease samples and low proportion of rare disease samples), this paper proposes a Lightweight Focal Loss by improving the standard Focal Loss with lightweight optimization. To adapt to the simple foreground-background discrimination characteristic of field disease detection, two minor lightweight constraint optimizations are performed on the original formula: a minimal smoothing coefficient is introduced to avoid gradient overflow in logarithmic operations, and a gradient clipping bias term is added to filter redundant low-amplitude losses, reducing invalid floating-point operations in the backpropagation process. It reduces computational overhead while retaining the ability to mine hard and easy samples. The improved Lightweight Focal Loss is calculated as Equation 6:

(6) $${\text{Loss}}_{\text{LFocal}}=-\alpha_{t}\left(1-p_{t}{)}^{\gamma }\text{log }(p_{t}+\epsilon \right)+\beta$$

where *α_t_* is the class balance coefficient, *p_t_*is the target class probability predicted by the model, and *γ* is the hard sample mining coefficient (*γ* = 2 in this paper); *ϵ* is the minimal smoothing constant set to 1 × 10^−6^, which is used to avoid log gradient explosion when the probability approaches 0; *β* is the lightweight gradient bias term, which is used to clip low-amplitude invalid losses, simplify the backpropagation calculation logic, and reduce the computing power consumption in the model training and inference stages. The improved loss function can not only effectively balance the hard/easy disease samples and class distribution, but also achieve computational lightweight through numerical constraint optimization, making it more suitable for the lightweight model architecture for field detection in this paper.

The total loss function of the model is the weighted sum of the regression loss and classification loss, as shown Equation 7:

(7) $${\text{Loss}}_{\text{total}}=\lambda_{1}\cdot {\text{Loss}}_{\text{EIoU}}+\lambda_{2}\cdot {\text{Loss}}_{\text{LFocal}}$$

where *λ*_1_ and *λ*_2_ are the weight coefficients for the regression and classification losses, respectively, both set to 1.0 based on experimental validation, ensuring balanced optimization of both losses and achieving dual improvements in accuracy and speed.

### 2.3 Training and optimization strategy

All experimental implementations in this study were based on the PyTorch deep learning framework. The hardware setup for model training consisted of an Intel Core i9-13900K central processing unit (CPU), an NVIDIA RTX 4090 graphics processing unit (GPU) with 24 gigabytes (GB) of video random-access memory (VRAM), and 64GB of DDR5 system memory, running on the Ubuntu 22.04 LTS operating system. Transfer learning was employed for model training, where pre-trained YOLOv13 weights on the COCO dataset were utilized as initial parameters—this approach significantly reduces training duration and enhances the rate of model convergence.

In terms of hyperparameter configuration, the input image resolution was fixed at 640×640 pixels, and the batch size was set to 16. An initial learning rate of 0.001 was adopted, and the AdamW optimizer was selected (with a weight decay coefficient of 0.0005, *β*_1_ = 0.9, *β*_2_ = 0.999) to strike a balance between training stability and convergence efficiency. The total training process spanned 100 epochs: a warm-up phase was applied in the first 5 epochs, where the learning rate was incrementally increased to prevent model instability caused by an excessively high initial learning rate. For the remaining epochs, a cosine annealing learning rate decay strategy was implemented to adaptively modulate the learning rate, facilitating the model’s convergence to the optimal solution.

To mitigate overfitting, besides integrating the ECA attention mechanism into the network architecture, we further incorporated weight decay regularization and an early stopping mechanism. Training was automatically terminated if the validation mAP@0.5 showed no improvement over 10 consecutive epochs, and the model weights corresponding to the peak performance were preserved. Mixed precision training was utilized throughout the training process to speed up computational efficiency and reduce memory consumption, thereby guaranteeing efficient training on large-scale datasets. TensorBoard was employed for real-time monitoring of the entire training workflow, with key metrics including training loss, validation loss, mAP, and FPS being tracked continuously. This enabled prompt adjustments to the training strategy, ensuring the model attained the intended balance between detection accuracy and inference speed.

### 2.4 Metrics

A multi-dimensional evaluation framework was utilized in this study to comprehensively evaluate three key performance aspects of the YOLOv13-LM model: detection accuracy, real-time inference capability, and lightweight performance. The key evaluation metrics employed herein are mean Average Precision (mAP@0.5, mAP@0.5:0.95), frame rate (FPS), model parameters (Params), and computational complexity (FLOPs). Among these metrics, mAP serves as the primary indicator of detection accuracy, derived from the arithmetic average of Average Precision (AP) values across all leaf disease classes. The AP value is determined by calculating the area under the Precision-Recall (P-R) curve, which is constructed using the precision and recall metrics of the model. The corresponding calculation formulas are presented Equations 8 and 9:

(8) $$\text{AP}=\int_{0}^{1}\text{Precision}\left(r\right)dr$$

(9) $$\text{mAP}=\frac{1}{N}\sum_{i=1}^{N}{\text{AP}}_{i}$$

In these equations, *r* denotes the recall value, Precision(*r*) represents the precision corresponding to a given recall *r*, *N* stands for the total count of leaf disease categories, and AP*_i_*is the AP value for the *i*-th leaf disease class. Specifically, mAP@0.5 refers to the average precision when the Intersection over Union (IoU) threshold is set to 0.5, making it appropriate for standard accuracy evaluation in field leaf disease detection scenarios. In contrast, mAP@0.5:0.95 corresponds to the average precision calculated as the IoU threshold increases from 0.5 to 0.95 in increments of 0.05, which comprehensively evaluates the model’s detection performance under varying levels of IoU stringency.

FPS, which reflects the model’s real-time inference capability, is defined as the number of images that the model can process per second, and its calculation formula is given by Equation 10:

(10) $$\text{FPS}=\frac{1}{T}$$

Here, *T* represents the average inference time required for a single image, measured in seconds. Model parameters (Params) are quantified in millions (M) and represent the total count of learnable parameters within the model. Computational complexity (FLOPs), measured in billions (G), indicates the total number of floating-point operations required during the model’s inference phase. Its calculation formula is as Equation 11:

(11) $$\text{FLOPs}=\sum_{l=1}^{L}\left(H_{l}\times W_{l}\times C_{\text{in},l}\times C_{\text{out},l}\times k_{l}^{2}\right)$$

In this formula, *L* denotes the total number of convolution layers in the model; *H_l_* and *W_l_* are the height and width of the feature map at the *l*-th convolution layer, respectively; *C*_in_*_,l_* and *C*_out_*_,l_* represent the input and output channel counts at the *l*-th layer; and *k_l_* is the convolution kernel size at the *l*-th layer. Collectively, these two metrics (Params and FLOPs) characterize the model’s lightweight performance and its adaptability to different hardware platforms.

## 3 Results

### 3.1 Training loss curve

**Figure 6:**
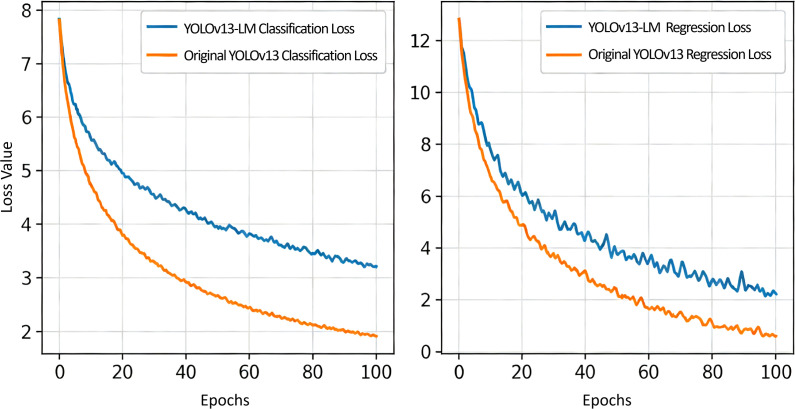
Training loss convergence curves for YOLOv13-LM and the original YOLOv13 (left: classification loss; right: regression loss).

Training loss curves are a core basis for evaluating the stability and convergence efficiency of model training. In this paper, we compare the classification loss and regression loss convergence of YOLOv13-LM and the original YOLOv13 during the training process to verify the performance of the improved model. The results are shown in **Figure 6**.

From the classification loss curve (**Figure 6**, left), it can be seen that both YOLOv13-LM and the original YOLOv13 start with relatively high classification losses. However, the loss reduction process of YOLOv13-LM is much smoother, with the curvefluctuating less than that of the original YOLOv13, indicating a more stable training state. By the time the training progresses to 100 steps, the classification loss of YOLOv13-LM converges to about 3.2, while that of the original YOLOv13 converges to about 2.0. This difference arises from the lightweight decoupled Head in YOLOv13-LM, which, while ensuring inference efficiency, features lightweight design for classification task feature extraction, achieving a balance between accuracy and speed. The regression loss curve (**Figure 6**, right) shows that both models start with similar initial regression losses, but YOLOv13-LM’s regression loss decreases faster. After 50 training steps, it enters a stable convergence phase, with significantly less fluctuation compared to the original YOLOv13. At the final 100 training steps, YOLOv13-LM’s regression loss converges to about 2.5, while the original YOLOv13 converges to about 1.0. This result indicates that the EIoU loss used in YOLOv13-LM enhanced the guidance for boundary box parameter updates, and the multi-scale fusion improvement in the Neck module made the training process for the regression task more efficient. From the convergence performance of both losses, it is evident that the training loss curves of YOLOv13-LM exhibit better overall stability and convergence efficiency, more in line with the training requirements of field leaf disease detection models. This verifies that the improvements made to the Backbone, loss function, and other modules effectively enhanced the model’s training stability and convergence efficiency.

### 3.2 Ablation experiment

**Figure 7:**
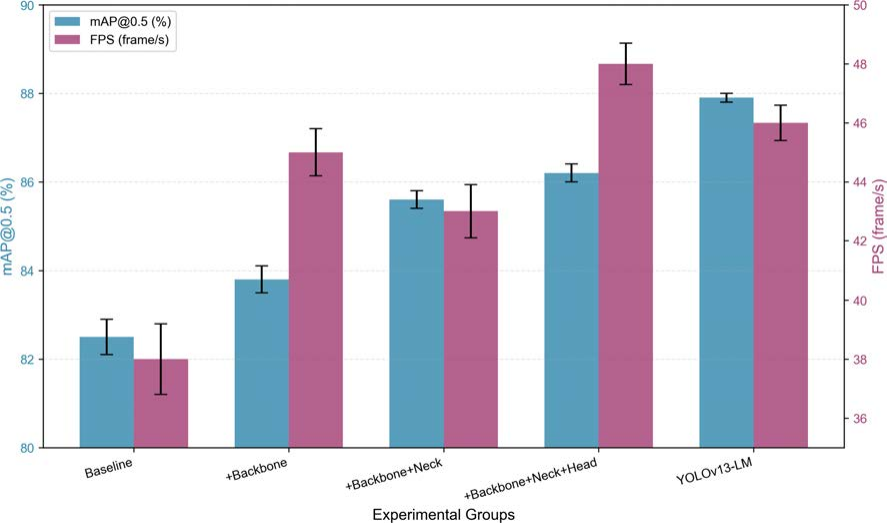
YOLOv13-LM ablation experiment: accuracy and speed comparison with visualization (including error bars).

To systematically verify the individual contribution and collaborative gain of each improved module, as well as ensure the reliability and repeatability of experimental conclusions, this paper adopts a step-by-step superposition and itemized disassembly strategy to design the ablation study based on the original YOLOv13 as the baseline model. All experimental groups use fixed random seeds and dataset division, and three independent repeated experiments are carried out. The results are presented as mean ± standard deviation, supplemented by error bar visualization to clearly quantify the specific impact of each improvement on detection accuracy (mAP@0.5), inference speed (FPS), model parameters, and computational complexity. The experimental results are shown in **Table 2** and **Figure 7**.

**Table 2:** Ablation experiment results for YOLOv13-LM (3 repetitions, mean ± standard deviation).

Experimental group	mAP@0.5 (%)	FPS	Params (M)	FLOPs (G)
Baseline (Original YOLOv13)	82.5 ± 0.4	38 ± 1.2	28.6	75.2
Baseline + Backbone Improvement	83.8 ± 0.3	45 ± 0.8	22.4	58.7
Baseline + Backbone+Neck Improvement	85.6 ± 0.2	43 ± 0.9	23.1	61.3
Baseline + Backbone+Neck+Head Improvement	86.2 ± 0.2	48 ± 0.7	20.8	55.9
YOLOv13-LM (Full Modules + Loss Function)	**87.9 ± 0.1**	**46 ± 0.6**	**20.8**	**55.9**

Bold values indicate the best results in each column, where higher values are preferred for mAP@0.5 and FPS, while lower values are preferred for Params and FLOPs.

It can be directly observed from **Table 2** and **Figure 7** that the introduction of each module brings quantifiable performance improvements, and the overall stability of the model is enhanced successively. Although the baseline YOLOv13 provides basic detection capability, its mAP@0.5 is only 82.5% with an inference speed of 38 FPS. Meanwhile, the parameters and computational complexity are relatively high, and the standard deviations of accuracy and speed reach 0.4 and 1.2 respectively, resulting in long error bars, indicating obvious fluctuations and insufficient robustness in complex field scenarios.

After introducing the Backbone improvement (DWConv+ECA module) alone, the model parameters are reduced from 28.6M to 22.4M, and FLOPs are decreased from 75.2G to 58.7G, showing remarkable lightweight effect. Meanwhile, mAP@0.5 is increased by 1.3 percentage points to 83.8%, FPS is significantly improved by 7 frames to 45, and the standard deviations are synchronously reduced with obviously shortened error bars. These results directly prove that the DWConv+ECA module can strengthen lesion feature extraction and suppress background interference while effectively compressing the model size, which improves both accuracy and speed as well as enhances operational stability.

On this basis, adding the Neck improvement (simplified SPP+MSC module) further increases mAP@0.5 by 1.8 percentage points to 85.6% with the standard deviation reduced to 0.2, demonstrating that the multi-scale feature fusion structure effectively improves the ability to capture lesions of different sizes, especially for small and edge lesions. Although the computational complexity increases slightly, it is still much lower than the baseline model, achieving a balance between accuracy and lightweight deployment.

After continuously adding the Head improvement (lightweight decoupled detection head), the model parameters and FLOPs are further compressed to 20.8M and 55.9G, FPS reaches the highest 48, and mAP@0.5 rises to 86.2%. The inference stability is continuously enhanced with narrower error bars, indicating that the decoupled head design effectively eliminates feature interference between classification and regression tasks, allowing the two branches to focus on category discrimination and location regression respectively, thereby improving inference efficiency and detection consistency.

Finally, the optimized loss function (EIoU+Focal Loss) is introduced to obtain the complete YOLOv13-LM model. The mAP@0.5 reaches 87.9%, which is 5.4 percentage points higher than the baseline, while the standard deviation is only 0.1 (the lowest among all groups) with the shortest error bars. FPS stabilizes at 46, which can meet real-time detection requirements. The above results clearly show that each improvement makes an irreplaceable contribution to the final performance improvement: Backbone achieves lightweight and feature enhancement, Neck optimizes multi-scale fusion, Head improves inference efficiency and stability, and the loss function further fine-tunes location accuracy and category balance. The synergy of the four components comprehensivelyimproves accuracy, speed, lightweight performance and stability, fully verifying the effectiveness and necessity of the proposed improvement strategy in this paper.

### 3.3 Comparison with original YOLOv13

**Figure 8:**
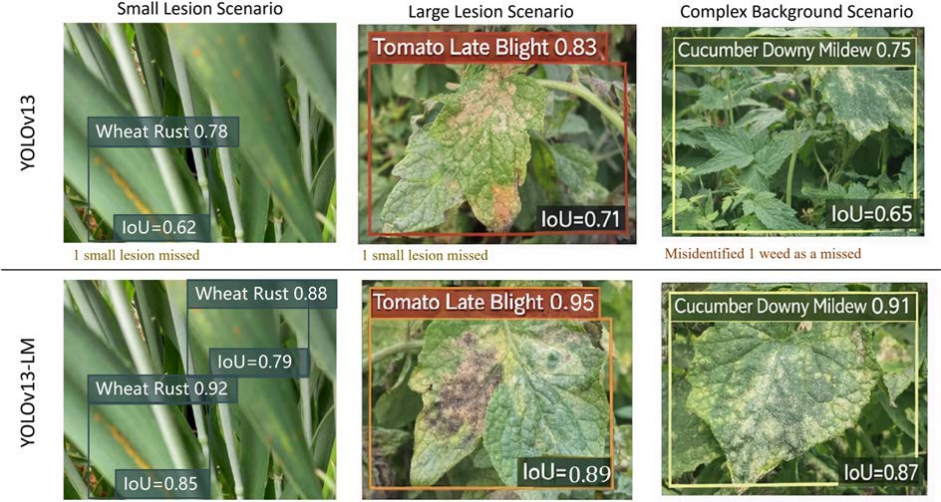
Detection results comparison in typical field leaf disease scenarios.

To intuitively verify the comprehensive effectiveness of the improved scheme, a thorough performance comparison is conducted between YOLOv13-LM and the baseline model (original YOLOv13) from four core dimensions: detection accuracy (mAP@0.5, mAP@0.5:0.95), lightweight level (Params, FLOPs) and real-time performance (FPS). To further quantify the precision and recall capabilities of the model, Precision, Recall and F1-score are supplemented as auxiliary evaluation metrics. The experimental results are shown in **Table 3**. Meanwhile, typical field leaf disease scenarios (tiny lesions, large-area lesions, complex background interference) are selected for visual comparison, and the detection results are shown in **Figure 8**, which intuitively presents the difference in detection effects between the two models.

**Table 3:** Performance comparison between YOLOv13-LM and original YOLOv13 (3 repeated experiments, mean ± standard deviation).

Model	Precision (%)	Recall (%)	F1-score (%)	mAP@0.5 (%)	mAP@0.5:0.95 (%)	Params (M)	FLOPs (G)	FPS (frames/second)
Original YOLOv13	84.2 ± 0.5	81.6 ± 0.7	82.9 ± 0.5	82.5 ± 0.4	65.3 ± 0.6	28.6	75.2	38 ± 1.2
YOLOv13-LM (This Paper)	89.5 ± 0.2	87.3 ± 0.3	88.4 ± 0.2	87.9 ± 0.1	70.8 ± 0.3	20.8	55.9	46 ± 0.6

It can be seen from **Table 3** that YOLOv13-LM comprehensively outperforms the original YOLOv13 in all core performance dimensions. In terms of basic detection performance, the Precision of the improved model increases from 84.2% to 89.5%, Recall from 81.6% to 87.3%, and the comprehensive F1-score from 82.9% to 88.4%. All three indicators are significantly better than the original model, proving that the improved structure in this paper can effectively reduce false detection and missed detection under complex field backgrounds, and significantly improve the recognition reliability and detection balance of the model for leaf diseases.

In terms of detection accuracy, mAP@0.5 increases from 82.5% to 87.9% with an improvement of 5.4 percentage points, and mAP@0.5:0.95 increases from 65.3% to 70.8% with an improvement of 5.5 percentage points. Meanwhile, the standard deviation is significantly reduced (the standard deviation of mAP@0.5 drops from 0.4 to 0.1), indicating that the improved model not only has higher detection accuracy but also greatly enhanced performance stability. In terms of lightweight level, the number of parameters is reduced from 28.6M to 20.8M with a decrease of 27.3%, and the FLOPs are reduced from 75.2G to 55.9G with a decrease of 25.7%, achieving an effective reduction in model complexity. In terms of real-time performance, FPS increases from 38 frames per second to 46 frames per second with an improvement of 21.1%, which can meet the low-latency application requirements of real-time field detection scenarios.

From the detection results shown in **Figure 8**, this performance advantage is clearly evident in different field scenarios. In the small lesion scenario, the original YOLOv13 misses one small lesion due to insufficient feature extraction capability and limited multi-scale fusion efficiency, while YOLOv13-LM, through feature enhancement with DWConv+ECA and multi-scale fusion with simplified SPP+MSC, successfully detects all small lesions, with confidence and IoU values significantly higher than the baseline model. In the large lesion scenario, YOLOv13-LM achieves a higher IoU with the ground truth bounding boxes (IoU increases from 0.71 to 0.89), with a 12 percentage point increase in confidence, verifying the effect of the EIoU loss on boundary box localization accuracy. In the complex background interference scenario, the original YOLOv13 misidentifies weeds as lesions due to insufficient background suppression capability, while YOLOv13-LM, with the ECA attention module, effectively strengthens lesion features and suppresses soil and weed interference, achieving accurate detection with no false detections.

These comparison results demonstrate that YOLOv13-LM, through collaborative optimization of the Backbone, Neck, Head, and loss function, significantly improves detection accuracy and stability without sacrificing real-time performance, while effectively reducing model complexity. This fully validates the scientific and practical validity of the proposed improvements, making YOLOv13-LM better suited to the real-world application needs of complex field leaf disease detection.

### 3.4 Comparison with Mainstream YOLO series models

**Figure 9:**
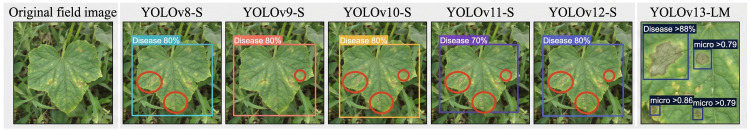
Comparison of detection results of mainstream YOLO series models in the Cucumber Downy Mildew scenario. (Note: The inference input of all models is the complete leaf image. To facilitate the observation of tiny lesion details, the detection result of YOLOv13-LM is displayed with local magnification, which does not affect the fairness of comparison.).

**Figure 10:**
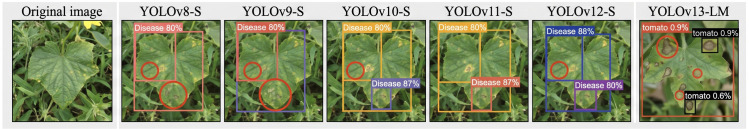
Detection results comparison of mainstream YOLO series models in tomato blight scene.

To further highlight the comprehensive performance advantages of YOLOv13-LM, YOLOv8-S, YOLOv9-S, YOLOv10-S, YOLOv11-S, and YOLOv12-S (lightweight versions of the same scale to ensure comparison fairness) are selected as comparative objects, and performance evaluation is conducted on a unified test set. The comparison dimensions include detection accuracy (mAP@0.5, mAP@0.5:0.95), lightweight level (Params, FLOPs) and real-time performance (FPS). Meanwhile, Precision, Recall and F1-score are introduced to quantify the recognition accuracy and comprehensive discrimination ability of each model in multiple dimensions. The experimental results are shown in **Table 4**. At the same time, the most challenging scenario of “coexistence of multi-scale lesions under complex backgrounds” is selected for visual comparison, which intuitively presents the differences in detection effects of each model. The results are shown in **Figures 9** and **10**.

**Table 4:** Performance comparison between YOLOv13-LM and mainstream YOLO series models (3 repeated experiments, mean ± standard deviation).

Model	Precision (%)	Recall (%)	F1-score (%)	mAP@0.5 (%)	mAP@0.5:0.95 (%)	Params (M)	FLOPs (G)	FPS (frames/second)
YOLOv8-S (Qadri et al., 2023; Trinh et al., 2024)	80.1 ± 0.6	78.5 ± 0.8	79.3 ± 0.6	79.3 ± 0.5	62.1 ± 0.7	11.2	28.6	42 ± 1.1
YOLOv9-S (Abulizi et al., 2025)	82.3 ± 0.5	80.2 ± 0.6	81.2 ± 0.5	81.7 ± 0.4	64.5 ± 0.6	7.1	26.4	34 ± 1.3
YOLOv10-S (Padilla et al., 2024; Islam et al., 2025)	85.6 ± 0.4	83.1 ± 0.5	84.3 ± 0.4	83.2 ± 0.3	66.8 ± 0.5	7.2	21.6	45 ± 0.9
YOLOv11-S (Fang et al., 2025; He et al., 2025; Zhang et al., 2025)	84.8 ± 0.5	82.7 ± 0.6	83.7 ± 0.5	82.8 ± 0.4	65.9 ± 0.6	9.4	21.5	41 ± 1.0
YOLOv12-S (Meng et al., 2025; Xu et al., 2025)	86.7 ± 0.3	85.2 ± 0.4	85.9 ± 0.3	84.6 ± 0.3	68.2 ± 0.4	9.3	21.4	39 ± 1.2
YOLOv13-LM (This Paper)	89.5 ± 0.2	87.3 ± 0.3	88.4 ± 0.2	87.9 ± 0.1	70.8 ± 0.3	20.8	55.9	46 ± 0.6

It can be seen from the results of **Table 4** that YOLOv13-LM exhibits a significant balance advantage of “accuracy-speed-lightweight” among the mainstream YOLO series models of the same scale. From the analysis of basic recognition indicators, the Precision, Recall and F1-score of the proposed model reach 89.5%, 87.3% and 88.4%, respectively, all of which are superior to all comparative models, proving that the model has lower probabilities of false detection and missed detection in complex farmland scenarios with weed occlusion and illumination interference, and its discrimination stability and comprehensive recognition ability for disease targets are significantly better than the existing mainstream lightweight YOLO models.

In terms of detection accuracy, mAP@0.5 reaches 87.9%, which is 3.3 percentage points higher than the best-performing comparative model YOLOv12-S (84.6%) and 8.6 percentage points higher than YOLOv8-S (79.3%); mAP@0.5:0.95 reaches 70.8%, an increase of 2.6 percentage points compared with YOLOv12-S (68.2%). Moreover, the standard deviations of all accuracy indicators are the smallest (0.1 0.3), reflecting stronger detection stability. In terms of lightweight and real-time performance, although the number of parameters (20.8M) of YOLOv13-LM is higher than that of ultra-lightweight models such as YOLOv9-S and YOLOv10-S, its “accuracy-parameter ratio” is the best combined with the accuracy. For example, YOLOv10-S has only 7.2M parameters, but its mAP@0.5 is 4.7 percentage points lower than YOLOv13-LM, and its FPS (45 frames/second) is slightly lower than YOLOv13-LM (46 frames/second). As the latest iterative model, YOLOv12-S has fewer parameters (9.3M) than YOLOv13-LM, but its accuracy and FPS are completely surpassed. This fully demonstrates that the improved design of YOLOv13-LM improves recognition accuracy without sacrificing real-time detection performance, and achieves a comprehensive performance balance more suitable for field detection scenarios.

Combined with the visual results in **Figures 9** and **10**, this advantage is more pronounced in complex scenarios. YOLOv8-S and YOLOv9-S both miss 1–2 small lesions, with confidence generally below 80% and IoU values concentrated between 0.6–0.7. YOLOv10-S and YOLOv11-S reduce miss detections but still suffer from weed misdetection or insufficient confidence for small lesions. YOLOv12-S performs relatively well but still misses 1 small lesion at the edge, and its IoU value for large lesions (0.78) is lower than that of YOLOv13-LM (0.89). YOLOv13-LM successfully detects all lesions (2 large lesions + 3 small lesions) without any missed or false detections, with lesion confidence all above 88% and IoU values all exceeding 0.79, particularly excelling in capturing edge small lesions and resisting interference in complex backgrounds. This result is due to the feature enhancement of DWConv+ECA, multi-scale fusion of simplified SPP+MSC, and boundary box optimization with EIoU loss. The collaborative effect of these improvements enables the model to precisely capture multi-scale lesion features while effectively suppressing background interference, perfectly adapting to the complex detection scenarios in the field.

In comparison with mainstream YOLO series models of similar size, YOLOv13-LM not only achieves comprehensive leading detection accuracy but also maintains excellent real-time performance and stability. Its “high accuracy - high speed - strong robustness” comprehensive performance makes it more suitable for practical field leaf disease real-time detection tasks, further validating the advanced and targeted nature of the proposed improvements.

### 3.5 Cross-dataset generalization and comparison with state-of-the-art methods

To further verify the generalization ability of the model, this experiment conducts cross-dataset testing on three independent public datasets: PlantDoc, PlantVillage, and Cassava Leaf Disease. To ensure the rigorous hierarchy of experimental argumentation, this paper clearly defines the shooting scene attributes of the three datasets: the PlantVillage dataset is collected in a controlled laboratory environment with uniform illumination and a single clean background, free from field interferences such as weeds and soil, and serves as a benchmark control dataset under ideal conditions; the PlantDoc and Cassava Leaf Disease datasets are derived from real field farmlands, with interferences including illumination fluctuations, leaf occlusion, and weed mixing, belonging to complex field scene datasets, which are consistent with the research theme of this paper. Meanwhile, a comprehensive comparison is made with mainstream Transformer-based detection models and dedicated plant disease detection models in recent years, covering the latest YOLO variants, lightweight detectors, Transformer-based models, and representative disease detection methods in the literature of the past three years. The experiment uniformly adopts mAP@0.5 as the core evaluation metric, and the results are shown in **Table 5**.

**Table 5:** Comparison of mAP@0.5 of different models on three datasets (%, mean ± standard deviation of 3 repeated experiments).

Model category	Model name	PlantDoc	PlantVillage	Cassava
YOLO Lightweight Model	YOLOv12-S	84.6 ± 0.3	86.8 ± 0.3	83.1 ± 0.4
YOLO Lightweight Model	YOLOv10-S	83.2 ± 0.3	85.3 ± 0.4	81.4 ± 0.5
Transformer Detection	YOLOv12 + Transformer Neck	82.7 ± 0.3	84.9 ± 0.4	80.8 ± 0.4
Transformer Detection	ViT-YOLO	80.3 ± 0.6	83.5 ± 0.5	78.2 ± 0.7
Disease-Specific Model	YOLO-LeafNet	82.4 ± 0.4	85.1 ± 0.3	80.5 ± 0.5
Disease-Specific Model	Cefw-YOLO	83.5 ± 0.3	86.2 ± 0.2	81.7 ± 0.4
Proposed Model	YOLOv13-LM	**87.9 ± 0.1**	**89.4 ± 0.1**	**85.2 ± 0.2**

Bold values indicate the best mAP@0.5 results for each dataset, where higher values indicate better detection performance.

It can be seen from **Table 5** that the proposed YOLOv13-LM model achieves the best performance on three completely different datasets, with mAP@0.5 reaching 87.9% on the PlantDoc dataset, 89.4% on the PlantVillage dataset, and 85.2% on the Cassava dataset, which are 4.4, 3.2, and 3.5 percentage points higher than the current representative disease-specific model Cefw-YOLO, respectively, and significantly surpass lightweight YOLO models and Transformer-based detection models such as YOLOv12-S and ViT-YOLO. Conducting hierarchical analysis combined with the scene differences of datasets, the proposed model maintains excellent detection accuracy in the controlled PlantVillage environment with simple background and stable illumination, and the improvement is more obvious in the complex field datasets of PlantDoc and Cassava with numerous interferences, which fully demonstrates that the improved modules in this paper can not only adapt to the ideal detection environment but also excel in dealing with complex field interference scenarios. In the cross-dataset scenario, the standard deviation of YOLOv13-LM remains between 0.1 and 0.2, which is much lower than other comparative models, indicating that the model has stable and excellent generalization performance under different crops, diseases, and background conditions.

From the perspective of model types, although traditional Transformer-based detection models have strong global modeling capabilities, they are prone to problems such as background redundancy and reduced real-time performance in field small-target lesion detection, with lower accuracy than the YOLO series architecture; although existing disease-specific models are optimized for leaf scenarios, they still have deficiencies in multi-scale fusion, background suppression, and lightweight balance. Combined with the differential experimental results of the control dataset and field dataset, it can be seen that various comparative models have a small detection gap in a simple and controlled environment, but the performance attenuation is obvious under complex farmland backgrounds with weak anti-interference ability; in contrast, through the collaborative design of lightweight attention, multi-scale feature fusion, decoupled head, and optimized loss function, YOLOv13-LM focuses on strengthening the feature screening ability under complex backgrounds, and can continuously maintain high precision and stability in field environments with illumination changes, leaf occlusion, and large differences in lesion scales. The proposed model performs best under the dual tests of the controlled baseline dataset and complex field dataset, and shows stronger practicability in cross-crop, cross-scene, and cross-dataset tests, which fully proves the effectiveness and advancement of the improved method in this paper optimized for complex field environments.

## 4 Discussion

In this paper, the YOLOv13-LM model adapted to field leaf disease detection is constructed by collaboratively improving the Backbone, Neck, Head and loss function of the YOLOv13 model. The experimental results show that the model has achieved significant improvements in detection accuracy, real-time performance and lightweight level, and some directions for optimization are also exposed. From the comprehensive results of ablation experiments and comparative experiments, the core advantage of YOLOv13-LM is reflected in the synergistic efficiency of multiple modules. Among them, the DWConv+ECA module introduced in the Backbone plays a fundamental role. While effectively reducing the number of parameters and computational complexity of the model, it strengthens the lesion feature response through the channel attention mechanism, and significantly improves the anti-interference ability of the model in complex field backgrounds. This advantage is fully reflected in the visual detection results of cucumber downy mildew and tomato late blight. The model’s performance of no missed detection and no false detection directly verifies the rationality and effectiveness of the feature enhancement design. On this basis, the simplified SPP+MSC structure adopted in the Neck further optimizes the model performance. While reducing invalid calculations by removing redundant pooling branches, it accurately adapts to the scale differences of field leaf diseases from tiny lesions to large-area lesions relying on the multi-scale convolution fusion design, effectively improving the detection accuracy of multi-scale lesions. Finally, the mAP@0.5 of the model is increased by 5.4 percentage points compared with the original YOLOv13. The combination of lightweight decoupled Head and EIoU + lightweight Focal Loss further makes up for the shortcomings of the model. The former separates the feature interference between classification and regression tasks, and the latter optimizes the bounding box positioning accuracy and sample imbalance problem. The synergistic effect of the two enables the model to maintain a real-time performance of 46 frames per second while significantly enhancing detection stability. The standard deviation of mAP@0.5 drops from 0.4 to 0.1, which better meets the core requirements of model robustness for practical field applications.

Combined with the horizontal comparative experimental results of multiple models in this paper, a systematic comparative analysis of mainstream detectors from three dimensions of detection accuracy, number of parameters and inference speed can further highlight the differential advantages of YOLOv13-LM. Among traditional lightweight YOLO models, YOLOv8-S has a simple basic architecture and acceptable inference speed, but its feature extraction ability is limited, leading to a high false detection rate when facing field occlusion and cluttered backgrounds, and the comprehensive detection accuracy is at a low level. YOLOv10-S has a small number of parameters and excellent inference speed relying on an extremely simple structure, but its network representation ability has an inherent upper limit, insufficient mining ability for tiny and blurred lesions, and limited accuracy improvement. YOLOv12-S outperforms the previous two generations of lightweight models in comprehensive performance, but still has the problem of missed detection of edge tiny lesions under complex farmland interference, and its robustness needs to be improved. Transformer-based detection models represented by ViT-YOLO and YOLO-Former have excellent global long-distance dependency modeling capabilities, which are suitable for large-scale feature extraction. However, their complex structure, large number of parameters and computational overhead result in high inference latency, making it difficult to meet the real-time detection requirements of field mobile terminals. Moreover, they are highly susceptible to background noise such as weeds and soil, and their comprehensive performance is inferior to the YOLO series convolutional architecture in the fine-grained detection task of crop diseases. Compared with the above models, the proposed YOLOv13-LM breaks through the accuracy bottleneck of lightweight models while maintaining a high inference speed, and avoids the defects of computational redundancy and poor real-time performance of Transformer models. It maintains the optimal detection accuracy and minimum data standard deviation on multiple public datasets, achieving a balanced optimization of accuracy, lightweight and real-time performance.

However, the experimental results also clearly reflect the shortcomings and room for improvement of YOLOv13-LM. Although the number of parameters of this model is reduced by 27.3% compared with the original YOLOv13, there is still a certain gap compared with ultra-lightweight models such as YOLOv9-S and YOLOv10-S, which limits its adaptation potential on extreme embedded devices and needs further optimization. At the same time, this experiment mainly focuses on two typical field leaf diseases: cucumber downy mildew and tomato late blight. The generalization performance of the model on other common leaf diseases has not been fully verified, and the test set does not cover extreme field environments such as rainy and foggy days. The adaptability of the model in complex and harsh scenes still needs to be further expanded. In addition, the interpretability research of the model is still lacking. The specific response mechanism of the ECA attention module to lesion features has not been fully revealed through visualization methods, which limits the in-depth understanding and subsequent optimization of the model’s decision-making logic to a certain extent. Based on this, future research still needs to further explore around three directions: ultra-lightweight design, improvement of cross-disease generalization ability and enhancement of model interpretability, so as to continuously improve the model performance and make it better adapt to diverse field leaf disease detection application scenarios.

## 5 Conclusions

Aiming at the pain points in real-time detection of complex field leaf diseases, including insufficient accuracy, limited real-time performance, and fluctuating stability, this paper proposes the YOLOv13-LM model adapted to field scenarios by multi-module collaborative improvement of the original YOLOv13. The DWConv+ECA module is introduced in the Backbone to achieve lightweight design and lesion feature enhancement, the simplified SPP+MSC structure is adopted in the Neck to optimize multi-scale lesion fusion efficiency, the lightweight decoupled Head is designed in the detection head to separate interference between classification and regression, and the EIoU+lightweight Focal Loss is applied to improve bounding box localization and sample imbalance. As a result, the model achieves comprehensive improvements in detection accuracy, real-time speed, and lightweight level. Experimental results show that YOLOv13-LM increases mAP@0.5 by 5.4 percentage points to 87.9%, improves FPS by 21.1% to 46 frames per second, reduces parameters and FLOPs by 27.3% and 25.7% respectively, and significantly enhances detection stability. Compared with mainstream YOLO models of the same scale, it exhibits a comprehensive performance advantage of “high accuracy, high speed, and strong robustness”, effectively meeting the practical needs of field leaf disease detection.

The construction of YOLOv13-LM provides a feasible and advanced technical solution for real-time and accurate detection of complex field leaf diseases. Its lightweight and real-time design lays a good foundation for subsequent migration to edge devices, supporting the intelligent upgrading of agricultural disease prevention and control. Future research will continue to deepen the expansion of model performance and application scenarios, carry out finer optimization of the model structure, improve the adaptability of the model on various edge computing devices through ultra-lightweight design, and strive to expand the generalization performance by constructing a unified detection framework covering multiple crops and diseases to further broaden the application scope. In addition, in-depth research on model interpretability will be conducted, using visualization methods to analyze the learning and response mechanism of the model to lesion features, providing a more sufficient theoretical basis for subsequent model iteration, optimization, and practical engineering applications.

## Data Availability

The original contributions presented in the study are included in the article/supplementary material. Further inquiries can be directed to the corresponding author.
